# Comparative transcriptome analysis of mulberry reveals anthocyanin biosynthesis mechanisms in black (*Morus atropurpurea* Roxb.) and white (*Morus alba* L.) fruit genotypes

**DOI:** 10.1186/s12870-020-02486-1

**Published:** 2020-06-17

**Authors:** Gaiqun Huang, Yichun Zeng, Ling Wei, Yongquan Yao, Jie Dai, Gang Liu, Zhongzheng Gui

**Affiliations:** 1 School of Biotechnology, Jiangsu University of Science and Technology, Zhenjiang, 212018 Jiangsu China; 2 Sericultural Research Institute, Sichuan Academy of Agricultural Sciences, Nanchong, 637000 Sichuan China; 3 Sericultural Research Institute, Chinese Academy of Agricultural Sciences, Zhenjiang, 212003 Jiangsu China

**Keywords:** Mulberry fruit, Anthocyanin, Biosynthesis, Transcriptome

## Abstract

**Background:**

To gain a better understanding of anthocyanin biosynthesis in mulberry fruit, we analyzed the transcriptome of the mulberry varieties Da 10 (*Morus atropurpurea* Roxb., black fruit) and Baisang (*Morus alba* L., white fruit).

**Results:**

We found that whereas Da 10 had high levels of cyanidin 3-*O*-glucoside (Cy), and pelargonidin 3-*O*-glucoside (Pg), Baisang contained only Cy, at low levels. Based on a comparative transcriptome analysis, we annotated more than 27,085 genes (including 1735 new genes). Genes that were differentially expressed between Da 10 and Baisang were detected at three stages of fruit development: S1 [4256 genes, 10 days post-anthesis (DPA)], S2 (5612 genes, 19 DPA), and S3 (5226 genes, 28 DPA). Anthocyanin biosynthesis was found to be associated with the expression of 15 core genes and 5 transcription factors. Relative to Baisang, Da 10 showed a significant upregulation of genes involved in the early stages (production of the intermediate compounds chalcone and dihydroflavonol) and late stages (production of Cy and Pg) of anthocyanin biosynthesis. Baisang showed a significant downregulation of the genes involved in the early stages of anthocyanin biosynthesis and overexpression of flavanone 3-hydroxylase (FLS), resulting in the generation of quercetin and/or myricetin but not anthocyanins.

**Conclusions:**

The biosynthesis of anthocyanin in mulberry fruit is initiated from the precursor, phenylalanine, and mediated by the upregulation of dihydroflavonol 4-reductase, anthocyanidin synthase, anthocyanidin 3-*O*-glucosyltransferase, and cyanidin-3-*O*-glucoside 2-*O*-glucuronosyltransferase, and downregulation of FLS to produce Cy and Pg.

## Background

Anthocyanins are a chemically diverse class of secondary metabolites belonging to the flavonoid group of plant compound. This versatile group of phenolic molecules, of which more than 635 have been identified to date, are responsible for the different colors (the blues, purples, and reds) of many fruits, seeds, and flowers [1]. Approximately 95% of all anthocyanins in nature are derived from six major anthocyanidins (aglycones), namely, cyanidin (Cy), pelargonidin (Pg), malvidin (Mv), peonidin (Pn), delphinidin (Dp), and petunidin (Pt) [2]. The presence and concentration of these molecules contribute to the pigmentation observed in plant tissues. For example, different blue shades in flowers are attributable to Dp, whereas reddish hues are due to Cy [3]. In addition to their roles as colorants, certain anthocyanins exert antiviral, antibacterial, and fungicidal activities [4, 5], and accordingly may play roles in protecting plants from infection by pathogenic microorganisms.

Anthocyanins typically show low toxicity to mammals and other vertebrates [6], and numerous studies have indicated that these flavonoids may have health-promoting properties, such as anti-inflammatory, anticarcinogenic, and cardioprotective activity, and efficacy in the control of obesity and alleviation of diabetes. The beneficial effects of anthocyanins, observed in cell line assays, animal studies, and clinical trials, are assumed to be associated with their strong antioxidant capacities [7]. These bioactivities have motivated research on anthocyanins, which are now among the most studied compounds in plant science, and anthocyanin biosynthesis pathways and regulatory mechanisms at the transcriptional level have been thoroughly investigated in many plant species [8, 9]. Although the anthocyanin biosynthesis pathways in maize, snapdragon, and petunia share many common reactions, they also show notable differences, given the large number of genes involved and their multiple complex interactions. On the basis of analyses of the components and content of anthocyanin in mulberry fruit, it has been established that the main component of anthocyanin in mulberry is Cy. However, Pg and Dp do not appear to be metabolized normally during mulberry anthocyanin synthesis, the reason for which remains unclear [10, 11]. These mechanisms ultimately affect the total anthocyanin content in plants, which vary substantially among plant species and even different cultivars of the same species [12].

The mulberry tree (*Morus* spp.) not only serves as a foodplant for silkworms (*Bombyx mori*) but has also been traditionally used as a medicinal plant in countries in eastern Asia. In China, mulberry fruit is used as a herbal medicine to protect against liver and kidney damage, improve eyesight, and strengthen the joints, and is also used for its anti-aging effects, and to treat sore throats, fever, hypertension, and anemia [13]. Mulberry fruit contains high amounts of anthocyanins, the differing levels of which are associated with the different colors of fruit [14]. At least 11 anthocyanins have been identified in mulberries, the main components of which are cyanidin-3-*O*-glucoside and cyanidin-3-*O*-rutinoside [15.16]. To date, studies on the biosynthesis of mulberry anthocyanins have mainly focused on the characterization and expression of a few selected genes involved in their synthesis [17.18]. However, the available information is limited, and consequently we have yet to gain a complete understanding of anthocyanin biosynthesis in mulberries. Approximately 15 mulberry species are recognized worldwide (*M. alba*, *M. nigra*, *M. multicaulis*, *M. atropurpurea*, *M. bombycis*, *M. mizubo*, *M. wittiorum*, *M. laevigata*, *M. cathayana*, *M. serrata*, *M. mongolica*, *M. notabilis*, *M. nigriformis*, *M. yunnanensis*, and *M. australis*), among which there are more than 100 mulberry fruit varieties. One of the most prominent differences distinguishing these varieties is the color of the fruit they produce, which can be black, pink, red, or white, and such natural variation among different mulberry types provides valuable opportunities for elucidating the pathways associated with anthocyanin biosynthesis.

In the present study*,* we focused our efforts in this regard on the two mulberry varieties Da 10 *(Morus atropurpurea* Roxb.) and Baisang (*Morus alba* L.) [15]. The leaves of Da10 are heart-shaped, relatively flat, emerald green, smooth, and slightly wrinkled, whereas those of Baisang are heart-shaped, either full or split, flat, dark green, smooth, and without wrinkles. Da 10 is characterized by large purple-black fruit, whereas in contrast, the fruits of Baisang are medium-sized and jade white in color. Previously, Yang et al. comparatively analyzed genetic structures among the 15 species and 4 subspecies of the traditionally classified mulberries. Clustering analysis showed that Nei’s genetic consistency coefficient between *M. atropurpurea* and *M. alba* was 0.9575, whereas the genetic distance was 0.0435, thereby indicating the close relationship between these two species [16].

For the purposes of the present study, we characterized and compared the transcriptional activity of Da 10 and Baisang, at different stages of fruit development defined by the number of days post-anthesis (DPA), namely, S1 (10 DPA), S2 (19 DPA), and S3 (28 DPA), using Illumina sequencing technique. The identities of the differentially expressed genes (DEGs) were determined based on reference to the Gene Ontology (GO) and Kyoto Encyclopedia of Genes and Genomes (KEGG) databases. Transcriptome data were validated by comparing RNA-Seq results with the findings of quantitative real-time PCR (qPCR). We also investigated the regulatory role of transcription factors in anthocyanin synthesis to gain a better understanding of the mechanisms underlying fruit color development in mulberry.

## Results

### Classes of color compounds in mulberry fruit

**Fig. 1:**
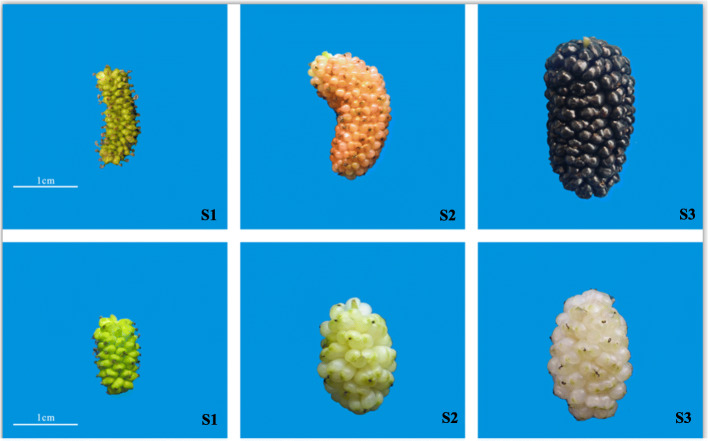
Representative images of mulberries from the two varieties Da 10 (upper row) and Baisang (lower row) at different developmental stages, S1 (10 days post-anthesis, DPA), S2 (19 DPA), and S3 (28 DPA)

To examine the biochemical factors underlying the lack of color development in white mulberry fruit, we compared the anthocyanin content of the two mulberry varieties Da 10 and Baisang at different stages of fruit development. In Da 10, fruit color changed from green, to purple, and subsequently to black at stages S1, S2, and S3, respectively (Fig. 1). For the Baisang variety, mulberries changed from green, to light green, and then to white at stages S1, S2, and S3, respectively (Fig. 1). The anthocyanin content in Da 10 comprised Cy and Pg. At S1, neither of these two anthocyanins were detected, whereas at stage 2, Cy and Pg levels were 23.533 μg/g and 4.197 μg/g, respectively. The levels of Cy and Pg increased during the ripening of Da 10, reaching 375.29 and 24.423 μg/g, respectively, at stage S3. In Baisang, Cy was the only anthocyanin detected, the levels of which ranged from 4.87 μg/g at S2 to 10.957 μg/g at S3 (Table 1). Thus, although the fruit of Baisang appear white, they contain low levels of Cy. None of the other anthocyanins Dp, Pn, and Mv were detected at any developmental stage in either of the two varieties.

**Table 1:** Anthocyanin content (μg/g) in mulberries from the two varieties, Da 10 (black) and Baisang (white), at different developmental stages, S1 (10 days post-anthesis, DPA), S2 (19 DPA), and S3 (28 DPA)

Sample	Delphinidin	Cyanidin	Pelargonidin	Peonidin	Malvidin
Da 10-S1	0.00	0.00	0.00	0.00	0.00
Da 10-S2	0.00	23.533 ± 3.371	4.197 ± 0.566	0.00	0.00
Da 10-S3	0.00	375.29 ± 2.851	24.423 ± 1.188	0.00	0.00
Baisang-S1	0.00	0.00	0.00	0.00	0.00
Baisang-S2	0.00	4.87 ± 0.896	0.00	0.00	0.00
Baisang-S3	0.00	10.957 ± 1.381	0.00	0.00	0.00

### Transcriptome sequencing, clustering, and functional enrichment of DEGs

As a consequence of quality filtering, we obtained 33.77–74.56 million high-quality 150-bp paired-end reads. Having removed rRNA reads, the sequencing reads were mapped to the *Morus notabilis* Schneid. reference genome (https://morus.swu.edu.cn/morusdb/), resulting in the annotation of more than 27,085 genes, including 1735 new genes (Table S1).

**Fig. 2:**
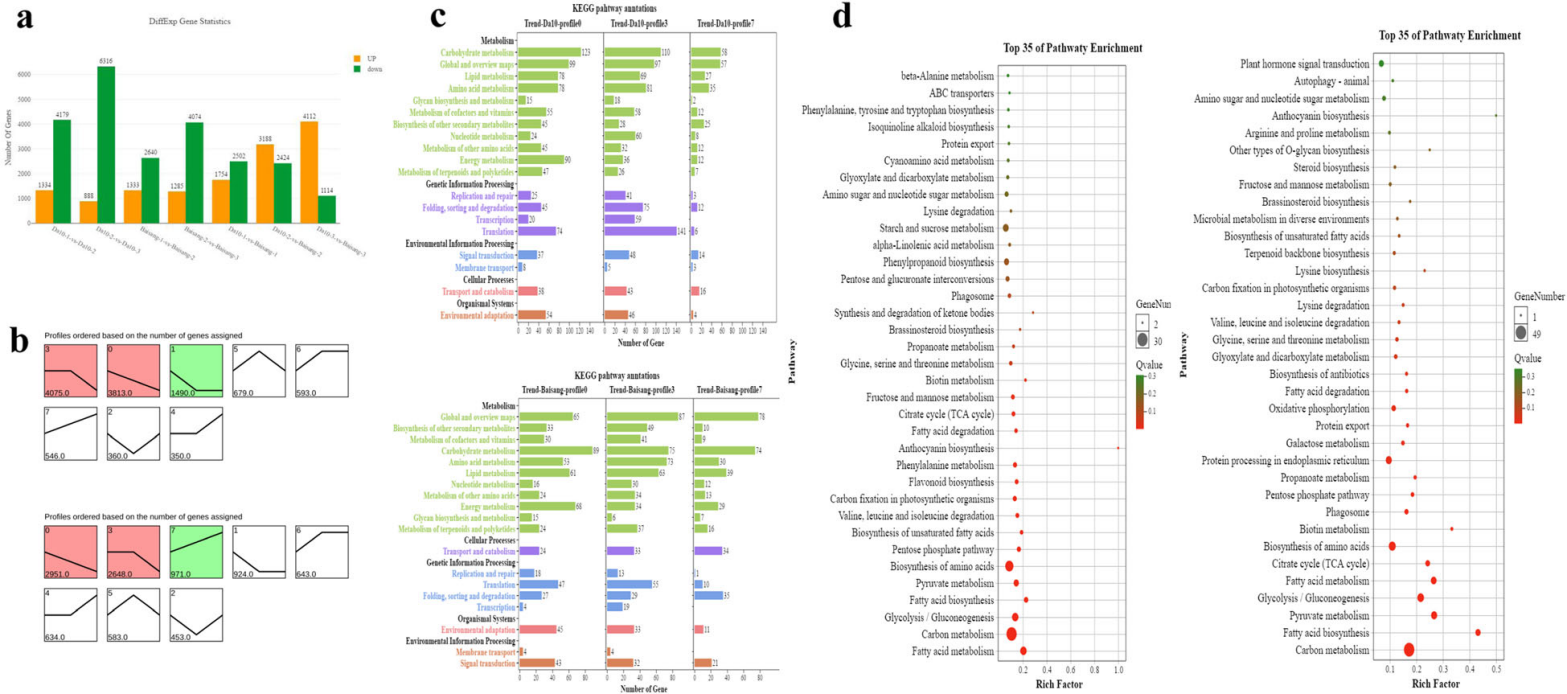
**Gene expression patterns and KEGG enrichment analyses of the two mulberry genotypes during fruit ripening.** (**a**) Differentially expressed gene analysis of the two mulberry genotypes. (**b**) Short Time-series Expression Miner (STEM) analysis of gene expression patterns in Da 10 (top) and Baisang (bottom). (**c**) KEGG annotation of Da10 (top) and Baisang (bottom) at three developmental stages: S1 (10 days post-anthesis, DPA), S2 (19 DPA), and S3 (28 DPA). (**d**) Pathway enrichment analysis of profile 7 (see text for details) in Da 10 (left) and Baisang (right)

DEGs were identified using the edgeR package (http://www.rproject.org/). Comparing the sequenced transcripts obtained at stages S1 and S2, we detected 5513 and 3973 DEGs in the fruit of Da 10 and Baisang, respectively, whereas our comparison between stages S2 and S3 enabled us to identify 7204 and 5359 in the fruits of Da 10 and Baisang, respectively. On the basis of the comparison between the two varieties, we detected 4256, 5612, and 5226 DEGs at developmental stages S1, S2, and S3, respectively (Fig. 2a).

To analyze DEG expression patterns, the expression data at different stage were normalized to 0, log_2_(v1/v0), and log_2_(v2/v0), and then clustered using Short Time-series Expression Miner software (STEM). DEGs in the fruit of the two varieties were clustered into eight profiles based on gene expression patterns. DEGs in profiles 0, 1, and 3 were significantly enriched in Da 10, whereas those in profiles 0, 3, and 7 were significantly enriched in Baisang (Fig. 2b). The DEGs in profiles 0 and 3 were downregulated in both genotypes. To examine the relationship between the enriched genes and metabolite accumulation, the DEGs in profiles 0, 3, and 7 were annotated for both varieties. Profile 7 contained 25 and 10 DEGs in Da 10 and Baisang, respectively, that are related to the biosynthesis of secondary metabolites. In profile 7, a larger number of the genes involved in carbohydrate, lipid, and energy metabolism were from Baisang (Fig. 2c). Pathway enrichment analysis showed that tyrosine, phenylalanine, tryptophan, phenylpropanoid, flavonoid, and anthocyanin biosynthesis were significantly enriched in profile 7 for Da 10, which indicates that anthocyanins were synthesized from the precursor phenylalanine, via the biosynthesis of phenylpropanoid, flavonoid, and anthocyanin. In Baisang, profile 7 contained genes involved in fructose and mannose metabolism, fatty acid metabolism, glycolysis/gluconeogenesis, and fatty acid biosynthesis (Fig. 2d).

### The anthocyanin biosynthesis pathway in mulberry fruit

Based on the data of transcriptome sequencing and DEG expression patterns to identify the key transcripts involved in anthocyanin metabolism, we compared the abundance of genes in the Da 10 and Baisang transcriptomes. We accordingly found that three genes involved in tyrosine, phenylalanine, and tryptophan biosynthesis, chorismate mutase (CM), arogenate dehydratase (PDT), and aspartate-prephenate aminotransferase (PAT), were upregulated in Da 10 but not in Baisang (Table S2). Other genes overexpressed in Da 10 relative to Baisang included those related to phenylpropanoid, flavonoid, and anthocyanin biosynthesis (Table S2, Fig. S1). We thus deduced that pathways involved in phenylalanine, tyrosine, tryptophan, phenylpropanoid, flavonoid, and anthocyanin biosynthesis are essential for the development of the Da 10 phenotype. Targeted metabolomics data indicated similar trends in the metabolites associated with these pathways.

**Fig. 3:**
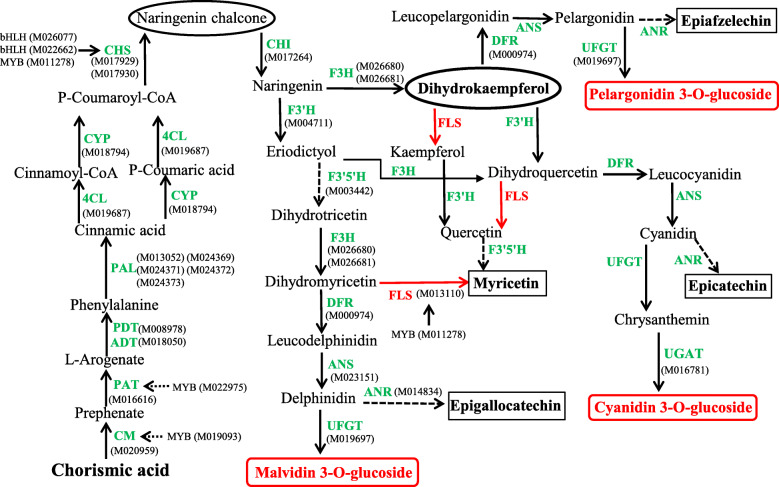
**A schematic representation of the anthocyanin biosynthesis pathway in mulberry fruit.** Genes colored green are involved in all anthocyanin biosynthesis pathways, whereas those colored red control myricetin synthesis

**Fig. 4:**
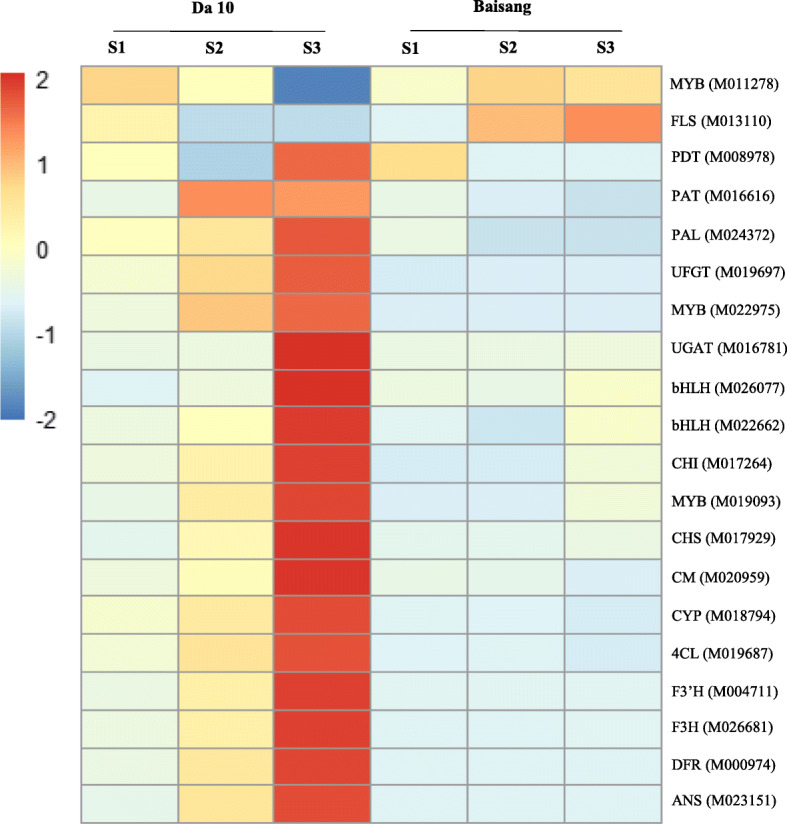
**Expression levels of the 15 core genes and five transcription factors involved in anthocyanin biosynthesis in mulberry fruit.** Each square represents the transcription level in the two mulberry varieties (Da 10 and Baisang) at different developmental stages, S1 (10 days post-anthesis, DPA), S2 (19 DPA), and S3 (28 DPA). Different color indicates differences in the level of gene expression, from high (red) to low (blue)

To obtain a global picture of the anthocyanin biosynthesis pathway in mulberry fruit, we compared the transcript levels of genes involved in anthocyanin synthesis, and the main related metabolic branches, in Da 10 and Baisang. Figure 3 presents a schematic representation of anthocyanin metabolism with its core metabolites and enzymes in mulberry fruit, highlighting the key steps that differ between the two mulberry varieties. Chalcone synthase (CHS) expression levels were found to be more than 40 times higher in black mulberries than in white fruit. A further gene upregulated in Da 10 is naringenin [2-oxoglutarate 3-dioxygenase (F3H, M026681)], the expression levels of which in black mulberries were found to be approximately 12 times higher than those in white fruit (Fig. 4 and Fig. S2). Expression at this level is expected to produce large amounts of another intermediate metabolite, dihydrokaempferol. The upregulation of these two pathways would enhance the supply of naringenin chalcone and dihydrokaempferol, thereby promoting the synthesis of anthocyanins in black mulberries (Fig. 3).

At the early stage of fruit development, both black and white mulberries showed similar levels of anthocyanins and patterns of gene expression. The presence of both Cy and Pg in black mulberries during the latter stages of development indicates that the anthocyanin biosynthesis pathway is probably induced at a point relatively far downstream of the late reactions catalyzed by enzymes such as dihydroflavonol 4-reductase (DFR), anthocyanidin synthase (ANS), or anthocyanidin 3-*O*-glucosyltransferase (UFGT). In contrast, the presence of myricetin in the white fruit indicates that the expression of flavonol synthase/flavanone 3-hydroxylase (FLS) was significantly upregulated (Figs. 3 and 4).

### Quantitation of related metabolites in the anthocyanin synthesis of mulberry fruit

On the basis of the annotated anthocyanin synthesis pathway in mulberry fruit, we performed an absolute quantitative analysis of metabolites, namely, 10 flavonoids and their precursors, including l-phenylalanine, naringenin, and cyanidin 3-*O*-glucoside, via the detection of targeted metabolites (Table 2). With respect to the phenylpropanoid biosynthesis pathway, we found that in Baisang, the content of l-phenylalanine and hydroxy-methoxycinnamate at the three stages of fruit development were higher than those in Da 10. Similarly, in the flavonoid biosynthesis pathway, the content of naringenin, dihydrokaempferol, eriodictyol, and dihydroquercetin at stages S1 and S2 in Baisang were higher than those in Da 10, whereas at stage 3, the content of these compounds was lower in Baisang than in Da 10. The content of dihydromyricetin at stage S3 was also found to be higher in Da 10 than in Baisang, although was not detected in either genotypes at stages S1 and S2. In the anthocyanin biosynthesis pathway of Baisang, we observed no significant changes in the content of cyanidin 3-*O*-glucoside or cyanidin 3-*O*-rutinoside in the three developmental stages, with only low level (25.10 μg/g and 13.01 μg/g, respectively) being detected at stage 3. Contrastingly, these two metabolites gradually increased in Da 10 concomitant with the development of fruit, reaching maximum levels of 9417.323 μg/g and 3227.725 μg/g, respectively, at stage 3. In the case of Baisang, whereas l-phenylalanine and other precursors were abundant, the amounts of naringenin and other intermediate products were considerably lower than those detected in Da 10 during fruit development.

**Table 2:** The absolute quantitative analysis of the intermediates in anthocyanin synthesis of mulberry fruit (μg/g)

Compounds	Class	Baisang-S1	Baisang-S2	Baisang-S3	Da 10-S1	Da 10-S2	Da 10-S3
L-Phenylalanine	Amino acids	811.31331 ± 36.43849	742.13025 ± 25.70976	443.71018 ± 23.07179	545.44409 ± 38.36563	253.50031 ± 15.51107	224.24296 ± 36.65830
Hydroxy-methoxycinnamate	Hydroxycinnamoyl derivatives	0.05897 ± 0.00184	0.07998 ± 0.00739	0.11390 ± 0.00284	0.04836 ± 0.00279	0.05797 ± 0.00778	0.09723 ± 0.00653
Naringenin	Flavanone	0.00515 ± 0.00051	0.01242 ± 0.00151	0.01036 ± 0.00166	0.00397 ± 0.00027	0.00472 ± 0.00039	0.03293 ± 0.00402
Dihydrokaempferol	Flavonol	0.00631 ± 0.00073	0.01445 ± 0.00054	0.02569 ± 0.00397	0.01084 ± 0.00092	0.00839 ± 0.00196	0.05662 ± 0.00244
Eriodictyol	Flavanone	0.02949 ± 0.00052	0.03769 ± 0.00506	0.06633 ± 0.00467	0.01500 ± 0.00027	0.01613 ± 0.00160	0.09237 ± 0.00604
Dihydroquercetin	Flavonol	0.19092 ± 0.01829	0.11948 ± 0.00843	0.60914 ± 0.06652	0.09268 ± 0.00079	0.02881 ± 0.00096	0.63948 ± 0.02406
Dihydromyricetin	Flavonol	N/A	N/A	0.01955 ± 0.00045	N/A	N/A	0.03062 ± 0.00149
Quercetin	Flavonol	0.35654 ± 0.00886	0.48267 ± 0.01911	0.66481 ± 0.04480	0.65661 ± 0.07946	0.41826 ± 0.01968	0.87541 ± 0.04971
Cyanidin 3-O-glucoside	Anthocyanins	23.62732 ± 0.18152	24.38825 ± 0.64472	25.10366 ± 0.39409	90.41854 ± 3.85833	599.96594 ± 93.58634	9417.32306 ± 266.24377
Cyanidin 3-O-rutinoside	Anthocyanins	13.32180 ± 0.07860	13.44661 ± 0.21209	13.01601 ± 0.24948	21.51326 ± 0.54634	377.16105 ± 35.91237	3227.72453 ± 76.47388

### Genes related to anthocyanin biosynthesis

The early genes include CM, arogenate dehydratase/prephenate dehydratase 6 (PDA), 4-coumarate-CoA ligase (4CL), phenylalanine ammonia-lyase (PAL), CHS, chalcone-flavonone isomerase (CHI), and F3′H, which contribute to the formation of dihydroflavonols. The late genes, DFR, ANS, UFGT, and cyanidin-3-*O*-glucoside 2-O-glucuronosyltransferase (UGAT), play roles in the generation of anthocyanins (Table S2, Fig. 3). In the present study, we identified 15 core genes and five transcription factors related to anthocyanin biosynthesis (Table S2), among which, 18 were upregulated. Only FLS and the MYB transcription factor (M011278) were downregulated in black mulberries relative to white fruit (Fig. 4). Notably, both early (CM, PAT, PAL, CYP, 4CL, and CHS) and late (DFR, ANS, UFGT, and UGAT) genes were significantly downregulated in white mulberries relative to black mulberries (Fig. 4). The outcome of the downregulation of UFGT would be a reduction in the synthesis of Mv and Pg, whereas downregulation of UGAT would lead to lower levels of Cy.

The formation of flavonols from dihydroflavonols is catalyzed by FLS, which acts on dihydrokaempferol, dihydroquercetin, and dihydromyricetin to produce kaempferol, quercetin, and myricetin, respectively. Compared with the black mulberry, we found that FLS expression was upregulated in the white mulberry (Fig. 4). It is conceivable that in the latter variety, the low levels of anthocyanin intermediates contribute to the downregulation of the core anthocyanin biosynthesis pathway genes, thereby inhibiting the synthesis of cyanidin, whereas the upregulation of FLS gene expression would block the synthesis of anthocyanins.

Our analysis of the mulberry transcriptome data resulted in the annotation of 1217 transcription factors, among which, 899 were found to be differentially expressed, including 78 MYB transcription factors, 83 bHLH (basic helix-loop-helix) transcription factors, and 31 MYB-related transcription factors. Most of the transcription factors involved in the regulation of genes related to the anthocyanin biosynthesis pathway belong to the MYB and bHLH families. In the present study, we found that the expression of transcription factors differed markedly between Da 10 and Baisang. Those in the MYB (M022975 and M019093) and bHLH (M026077 and M022662) families involved in anthocyanin biosynthesis were upregulated in black mulberries relative to white mulberries, whereas MYB (M011278) showed an inconsistent expression pattern.

### Validation of the expression of core genes and transcription factors using qPCR

In order to verify the expression levels of core genes and transcription factors related to mulberry anthocyanin synthesis, we selected the Morus010170 gene as a reference gene, based on its stable expression in all stages of fruit development in both genotypes. We then validated the expression of 19 genes using qPCR (Table 3, Fig. S2). The expression levels of these 19 genes at different developmental stages were consistent with those determined using RNA-Seq. All 19 genes (particularly CHS, F3′H, F3H, DFR, UFGT, and ANS) were found to be expressed at considerably higher levels in Da 10 than in Baisang. Moreover, in all samples, gene expression was significantly higher at stages S2 and S3 than at stage S1. These results accordingly indicate that these 19 genes are involved in the biosynthesis of Cy and Pg via the mulberry anthocyanin biosynthesis pathway. Furthermore, we speculate that the downregulated expression of related genes (DFR, ANS, UFGT, and UGAT) and the upregulated expression of FLS may contribute to the suppression of anthocyanin synthesis in Baisang fruit.

**Table 3:** Expression levels of core genes and transcription factors involved in anthocyanin biosynthesis in mulberry fruit measured by qPCR

Gene_ID	Da 10-S1	Da 10-S2	Da 10-S3	Baisang-S1	Baisang-S2	Baisang-S3
Morus020959	1.18222±0.15889^bc^	1.88258±0.25197^b^	7.77758±1.14978^a^	0.72409±0.03334^cd^	0.62440±0.00670^cd^	0.18361±0.01232^d^
Morus016616	1.06168±0.06172^d^	2.73895±0.04598^b^	2.96170±0.12892^a^	1.35136±0.14360^c^	0.74855±0.00177^e^	0.51202±0.02061^f^
Morus008978	2.08985±1.04272^b^	0.55526±0.03775^c^	3.98063±0.54728^a^	4.43413±1.67624^a^	1.39548±1.28161^bc^	1.24409±0.13577^bc^
Morus024372	1.16249±0.18139^c^	2.91039±0.27492^b^	8.06253±0.69500^a^	0.21913±0.02372^d^	0.21216±0.02373^d^	1.03741±0.02173^c^
Morus019687	1.22060±0.20707^c^	3.37482±0.08522^b^	6.79941±0.43346^a^	0.36948±0.04739^d^	0.34017±0.02607^d^	0.10043±0.00688^d^
Morus018794	1.12094±0.11790^c^	2.51933±0.08317^b^	5.15479±0.27124^a^	0.33514±0.01251^d^	0.19838±0.01246^d^	0.03483±0.00088^d^
Morus017929	1.25044±0.23356^c^	60.05397±6.67163^b^	204.42567±16.72710^a^	0.24663±0.05032^c^	0.70137±0.11956^c^	9.38162±1.11148^c^
Morus017264	1.16249±0.18139^c^	2.91039±0.27492^b^	8.06253±0.69500^a^	0.21913±0.02372^d^	0.21216±0.02373^d^	1.03741±0.02173^c^
Morus004711	1.11432±0.14113^c^	9.52750±1.83368^b^	19.26487±0.73340^a^	0.01153±0.00292^c^	0.03839±0.00177^c^	0.16674±0.02614^c^
Morus026681	1.18738±0.19339^c^	4.39685±0.33282^b^	12.12864±1.21909^a^	0.12576±0.00747^d^	0.17875±0.00741^d^	0.38565±0.01410^cd^
Morus000974	1.16340±0.23216^c^	6.59875±0.63817^b^	17.33984±1.65727^a^	0.00354±0.00181^c^	0.01215±0.00202^c^	0.00158±0.00047^c^
Morus023151	1.16012±0.17505^c^	11.52419±0.24537^b^	27.29133±1.82231^a^	0.00568±0.00231^c^	0.01615±0.00323^c^	0.00420±0.00125^c^
Morus013110	1.17889±0.16729^bc^	0.20182±0.01650^d^	0.44767±0.15683^d^	0.94758±0.04242^c^	1.97049±0.17624^ab^	2.02151±0.17312^a^
Morus019697	1.04230±0.08904^c^	3.33870±0.05326^b^	6.15405±0.27407^a^	0.00067±0.04000^d^	0.00039±0.04000^d^	0.00024±0.00007^d^
Morus022662	0.98671±0.01461^cd^	1.18920±0.14632^bd^	3.86468±0.45449^a^	1.15804±0.06608^b^	0.67690±0.00231^c^	1.37798±0.04622^b^
Morus026077	0.94035±0.05657^c^	1.69939±0.32301^b^	8.28561±1.13372^a^	2.35982±0.52593^b^	0.77495±0.12562^c^	2.18711±0.25806^b^
Morus019093	1.15571±0.15154^b^	5.54870±0.14925^b^	14.94315±0.25314^a^	0.04933±0.01419^d^	0.07291±0.00429^d^	1.44544±0.27548^c^
Morus022975	1.43737±0.38951^c^	5.83052±0.18116^b^	8.98590±1.54734^a^	0.14698±0.00992^d^	0.03833±0.00339^d^	0.01377±0.00210^d^
Morus011278	0.96787±0.02825^d^	0.81453±0.09266^e^	0.34772±0.00691^f^	2.83443±0.08113^a^	2.47529±0.12662^b^	1.97841±0.11099^c^

Note: In the same row values with different small letter superscripts mean significant difference (*P*<0.05).

## Discussion

### Anthocyanin compounds and transcriptome analysis of mulberry fruit

We investigated the biosynthesis of anthocyanins in mulberry by comparison of their levels in the mulberry varieties Da 10 (black fruit) and Baisang (white fruit). The anthocyanin content in Da 10 comprised Cy and Pg, whereas Cy was the only anthocyanin detected with the low level in Baisang. The presence in mulberries of the two anthocyanins Cy and Pg, both of which are cyanidin derivatives, is consistent with the findings of earlier studies [17, 18]. These results confirm, at the metabolite level, the genotype-dependent difference in the accumulation of anthocyanins in mulberry varieties, as well as the association between fruit color and anthocyanin levels. In this regard, Cy and Pg appear to be the main anthocyanins determining the color of mulberry fruit. Once formed, the unstable Cy would be converted to the colorless epicatechin, which would permanently prevent the formation of stable color pigments via later glycosylation and other reactions.

Based on a comparative transcriptome analysis, we annotated more than 27,085 genes, including 1735 new genes. Genes that were differentially expressed between Da 10 and Baisang were detected 4256, 5612, and 5226 DEGs at developmental stages S1, S2, and S3, respectively (Fig. 2a).

### The anthocyanin biosynthesis pathway in mulberry fruit

Anthocyanin synthesis in the leaves, fruits, and flowers of *Arabidopsis thaliana*, grape, and hyacinth has been shown to be associated with three secondary metabolic pathways, namely, the phenylpropanoid, flavonoid, and anthocyanin biosynthesis pathways [19–21]. The lack of color development in white mulberries would require a complete blockage of the anthocyanin biosynthesis pathway, which presumably occurs prior to the formation of Dp and Cy [22]. In this study, three genes involved in tyrosine, phenylalanine, and tryptophan biosynthesis, CM, PDT, and PAT, were upregulated in Da 10 but not in Baisang (Table S2). CHS and F3H expression levels were found to be more than 40 times and 12 times higher respectively, in black mulberries than in white fruit (Fig. 4 and Fig. S2). It is assumed that such overexpression would lead to a large production of the intermediate metabolite naringenin chalcone in black mulberry. CHS catalyzes the first reaction of anthocyanin biosynthesis, and subsequently contributes to the formation of chalcone (intermediate), which is the primary precursor of flavonoids [23]. Consequently, when chalcone synthesis is constrained, both anthocyanin production and that of nearly all other flavonoids, is affected [24]. DFR, ANS, UFGT and FLS hydroxylation reactions during anthocyanin biosynthesis pathway are essential to the formation of different types of anthocyanins, and eventually produce different colors. Indeed, anthocyanin accumulation is associated with the expression of the genes encoding the enzymes that participate in these reactions [25–27].

Quantitation of related metabolites in the anthocyanin synthesis of mulberry fruit showed that the content of cyanidin 3-*O*-glucoside and cyanidin 3-*O*-rutinoside in Baisang were substantially lower than those in Da 10. In both genotypes, quercetin accumulates at stage S1; however, whereas it gradually increased in Baisang during the latter stages of fruit development, it was found to decrease in Da 10, thereby indicating that the upregulated expression of the *FLS* gene promotes the conversion of dihydroquercetin to quercetin, and further inhibits anthocyanin synthesis.

### Expression of core genes and transcription factors in mulberry fruit

Genes that regulate the anthocyanin biosynthesis pathway are generally classified as early and late genes [28]. In this study, both early (CM, PAT, PAL, CYP, 4CL, and CHS) and late (DFR, ANS, UFGT, and UGAT) genes were significantly downregulated in white mulberries relative to black mulberries. And, FLS expression was upregulated in the white mulberry (Fig. 4). Chalcone synthase is a plant-specific polyketide synthase that plays a key role in flavonoid biosynthesis. In strawberries, CHS is expressed not only in the petals but also in the fruit (in which its transcripts are abundant) [29] during strawberry ripening, and upregulation of the CHS, F3′H, DFR, and UFGT genes corresponds to an increase in fruit enzymatic activity [30], resulting in the accumulation of anthocyanin at the ripe red stage. Similar observations have been made with respect to ANS expression and anthocyanin accumulation in *Allium cepa* [31], *Duchesnea indica* [32], and *M. alba* [17, 33].

The functions of MYB transcription factors in the regulation of genes associated with anthocyanin accumulation have been reported in several plant species, including rice (*Oryza sativa*) [34], *A. thaliana* [35], maize (*Zea mays*) [36], petunia (*Petunia hybrida*) [37], grapevine (*Vitis vinifera* L.) [38], apple (*Malus domestica*) [39], and poplar (*Populus tremuloides*) [40], based on genetic and molecular analyses. The bHLH proteins form the second largest family of transcription factors in plants, in which they play an important role of metabolic, physiological, and developmental processes [41]. The present results showed that the expression of transcription factors differed markedly between Da 10 and Baisang. MYB (M022975 and M019093) and bHLH (M026077 and M022662) were upregulated in black mulberries relative to white mulberries, whereas MYB (M011278) showed an inconsistent expression pattern.

## Conclusion

The biochemical and molecular findings of this study provide a more comprehensive insight into those mechanisms underlying the synthesis and accumulation of anthocyanins in different mulberry genotypes. We observed substantial increases in levels of the two anthocyanins Cy and Pg during late ripening of Da 10 fruits, whereas in Baisang, only Cy was detected, although only at low levels and exclusively during the late stage of fruit development. The high content of anthocyanins in black mulberries was associated with an upregulation of genes involved in the initial steps of anthocyanin synthesis (CM, CHS, and CHI), which produce the intermediates, chalcone and dihydroflavonol. The expression of genes that ultimately yield Cy, Pg, and Mv during the latter steps of anthocyanin synthesis (DFR, ANS, UFGT, and UGAT) was also upregulated in black mulberries. In contrast, the low anthocyanin content in white mulberries can be attributed primarily to the downregulation of early genes (MYB, bHLH, CHS, and CHI), and an upregulated expression of FLS, leading to the generation of quercetin and/or myricetin.

## Methods

### Plants and reagents

Fruit samples from the two mulberry genotypes [black fruit (Da 10 variety) and white fruit (Baisang variety)] (Fig. 1) were collected from the mulberry garden of the Sericultural Research Institute, Sichuan Academy of Agricultural Sciences, China. Nine plants of each variety were randomly selected, and 20 fruits from each plant were collected at each of the three assessed developmental stages, which were defined as follows: S1 (green, 10 DPA), S2 (semi-mature, 19 DPA), and S3 (mature, 28 DPA). Fruits harvested at the same stage in the same variety were mixed and divided into three replicate groups, and then frozen in liquid nitrogen and immediately stored at − 80 °C for subsequent anthocyanin determination and transcriptome analysis.

Anthocyanin analytical standards (for cyanidin, delphinidin, peonidin, pelargonidin, and malvidin) were purchased from Sigma-Aldrich (St. Louis, MO, USA). Iodomethane, dimethyl carbonate, dimethyl phosphate, and acetonitrile were provided by Sangon Biotech (Shanghai, China). Other reagents used were of analytical grade.

### Determination of mulberry anthocyanin levels

The extraction and determination of anthocyanins from mulberry fruit were performed using a previously described method [42]. Anthocyanin extraction was carried out using acidified methanol (methanol and 1.0 N HCl, 85:15, v/v), assisted by ultrasonic disruption. The extracts were concentrated by evaporation at 50 °C using a rotary evaporator, and were then re-dissolved in acidified methanol. Individual anthocyanins were separated and quantified using a high-performance liquid chromatography (HPLC) system (1260 Infinity II LC, Agilent Technologies Inc., CA, USA), coupled to a dual wavelength ultraviolet–visible detector and a data acquisition system (Millennium Chromatography Manager version 2.15.01; Waters Corporation, MA, USA). A reversed-phase chromatography column (Supelcosil LC-18-dB, 25 cm × 4.6 mm i.d.; Agilent) was used at room temperature.

### RNA extraction and sequencing

Total RNA isolation was performed using an RN09-EASY spin plus Plant Kit (Aidlab Biotech, Beijing, China) based on a previously described method [43]. The integrity of the extracted RNA was verified by agarose gel electrophoresis, and the concentration was measured using a 2100 Bioanalyzer (Agilent Technologies, CA, USA). cDNA libraries were constructed for each sample using enriched mRNA. High-quality RNA was used for subsequent RNA sequencing, which was performed using the HiSeq™ 2500 platform (Illumina, San Diego, CA, USA) at Guangzhou Gene Denovo Biotechnology Co. Ltd. (Guangzhou, China).

### Normalization and identification of DEGs

Sequencing reads were mapped to the reference sequences of *M. notabilis* using SOAPaligner/SOAP2 [44]. Gene expression levels were measured as fragments per kilobase of exon model per million reads mapped (FPKM), with the longest transcript being used to calculate the FPKM.

DEGs were identified by comparing expression levels of the transcripts of Da 10 and Baisang at the same developmental stage. For the correction of multiple testing, the false discovery rate (FDR) was analyzed for adjustment of the p-value threshold [45]. To assess changes in gene expression patterns during fruit ripening within the two genotypes, expression pattern analysis was performed using Short Time-series Expression Miner (STEM, version 1.3.8) [46] based on all the DEGs of Da 10 and Baisang mulberries.

### Annotation and GO and KEGG functional classification

All expressed genes were functionally annotated using the following databases: the NCBI nonredundant protein database (Nr), the Kyoto Encyclopedia of Genes and Genomes (KEGG), the Clusters of Orthologous Groups of proteins database (COG), and the Swiss-Prot database. For the purposes of annotation, we used BLASTX with an e-value cutoff of 1e-5 in Blast2GO [43]. For transcript sequences, the matched protein with the highest similarity rate was determined to be the optimal annotation.

Gene Ontology (GO) classification was performed for the upregulated genes in Da 10 and Baisang using Web Gene Ontology Annotation Plot (WEGO) [43], and a χ^2^ test was used to identify GO terms over-represented (or under-represented) in one variety compared with the other. To identify KEGG pathways, the numbers of up- and downregulated genes in each genotype were compared with those in the reference set.

### Metabolite validation and expression analysis

To analyze the accumulation pattern of metabolites during mulberry fruit ripening, we conducted targeted metabolomics for absolute quantitative analysis of a number of anthocyanin-related substances. LC-ESI-MS/MS system (HPLC: Shim-pack UFLC SHIMADZU CBM30A system; MS: Applied Biosystems 6500 Q TRAP) was used to analyze extracted samples. LIT and triple quadrupole (QQQ) scans were attained using a triple quadrupole-linear ion trap mass spectrometer (Q TRAP), and an API 6500 Q TRAP LC/MS/MS system, equipped with an ESI Turbo Ion-Spray interface, operating in the positive ion mode and controlled by Analyst 1.6 software (AB Sciex). With the analytical conditions, the HPLC column used was Waters ACQUITY UPLC HSS T3 C18 (1.8 μm, 2.1 mm × 100 mm); The mobile phase was composed of two solvents, 0.04% acetic acid (solvent A): acetonitrile with 0.04% acetic acid (solvent B). The linear gradient elution program was set as: 100% A for 0 min, 100–5% A for 12.0 min, 5–95% A for 12.1 min, and maintained at 5–95% A for 15.0 min. The flow rate was 0.40 mL/min; temperature was 40 °C and injection volume was 2 μL. The eluent was connected to an ESI-triple quadrupole-linear ion trap (Q TRAP)-MS. To ensure reproducibility and reliability, at least three independent biological replicates were analyzed for each sample using the LC-ESI-MS/MS system.

### Validation and expression analysis

A set of genes predicted by RNA sequencing to be related to color development were selected for qPCR assays. First-strand cDNA was synthesized using a FastQuant RT Kit (TIANGEN Biotech, KR106, Beijing, China). Gene-specific primers for qPCR were designed using Primer Premier software (Table S3). The Morus010170 gene was used as an internal control to normalize gene expression. The qPCR assays were performed using SYBR Premix *Ex TaqII* (Tli RNaseH Plus; Takara Bio, Shiga, Japan) in a LightCycler 480 instrument (Roche). To ensure reproducibility and reliability, three biological replicates were performed for each gene. We performed regression analysis between the qPCR and RNA sequencing data for 20 genes of the two genotypes at the three fruit-ripening stages using R version 3.1.3 (http://cran.r-project.org/).

### Statistical analysis

All the tests were repeated three times. The data was examined via analysis of variance (one-way ANOVA). The statistical analysis was performed using PASW Statistics 18.

## Supplementary information

**Additional file 1: Table S1.** Transcriptome sequence numbers of mapped reads from the two mulberry genotypes at three developmental stages.

**Additional file 2: Table S2.** Core genes related to anthocyanin biosynthesis in mulberry fruit.

**Additional file 3: Table S3.** Primers used for qPCR.

**Additional file 4: Figure S1.** Biosynthesis pathway in mulberry fruit. a. Phenylalanine, tyrosine, and tryptophan biosynthesis. b. Phenylpropanoid biosynthesis. c. Flavonoid biosynthesis. d. Anthocyanin biosynthesis.

**Additional file 5: Figure S2.** Expression levels of the core genes and transcription factors involved in anthocyanin biosynthesis in mulberry fruit, measured by qPCR.

## Data Availability

The datasets generated and/or analyzed during the current study are available at NCBI project PRJNA636910 (https://www.ncbi.nlm.nih.gov/bioproject/PRJNA636910) with accession number SRP265866. Any reasonable requests are available from the corresponding author.
