# Supplementary material for: Comparative transcriptome analysis of mulberry reveals anthocyanin biosynthesis mechanisms in black (Morus atropurpurea Roxb.) and white (Morus alba L.) fruit genotypes
Source: BMC Plant Biol. 2020 Jun 17;20:279. doi: 10.1186/s12870-020-02486-1 (PMC7301479; doi:10.1186/s12870-020-02486-1)
Supplement: Supplementary file 1 — Additional file 1: Table S1. Transcriptome sequence numbers of mapped reads from the two mulberry genotypes at three developmental stages. [file 12870_2020_2486_MOESM1_ESM.pptx]

## Slide 1
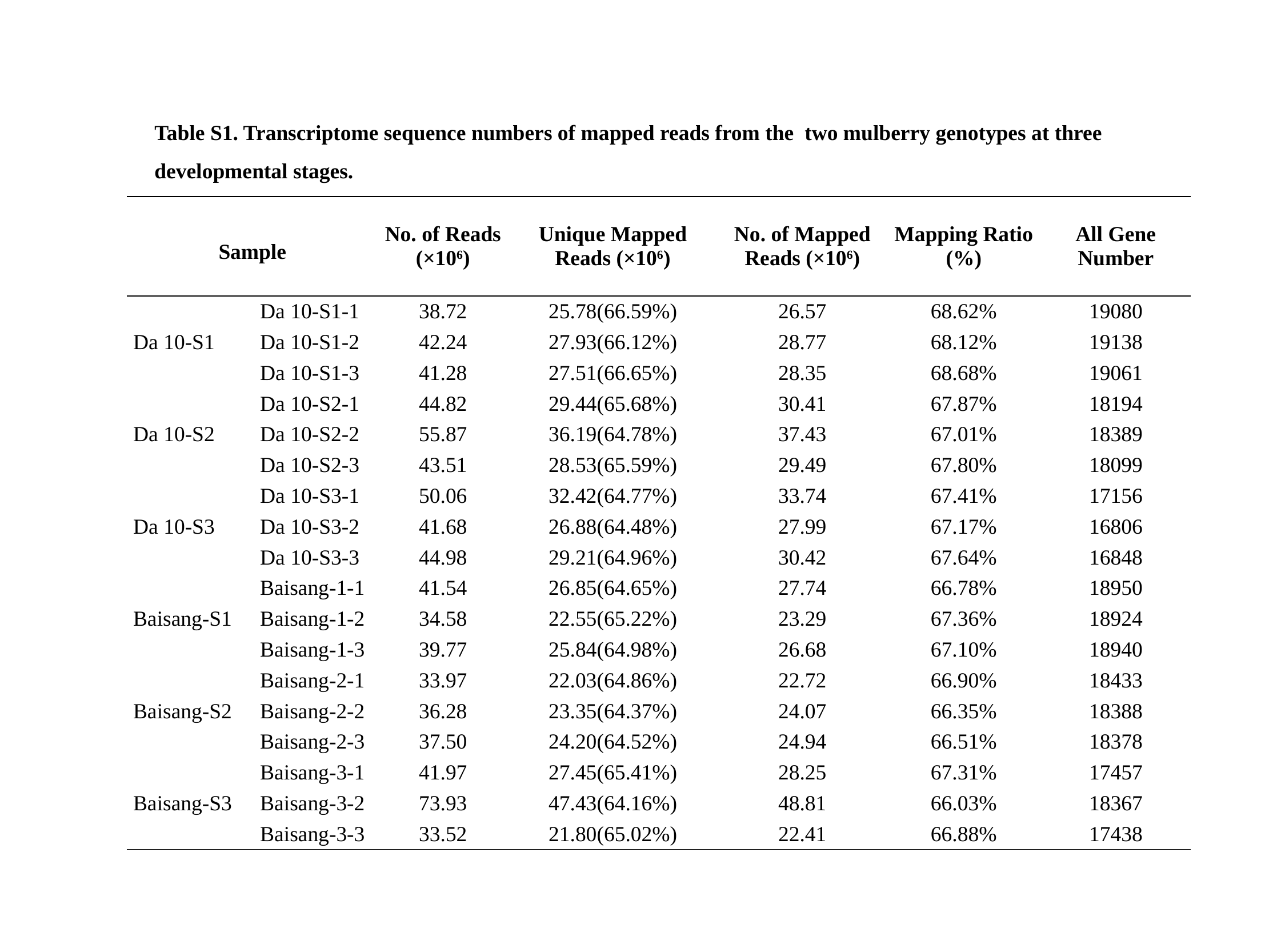

Table S1. Transcriptome sequence numbers of mapped reads from the two mulberry genotypes at three developmental stages.
| Sample | | No. of Reads (×106) | Unique Mapped Reads (×106) | No. of Mapped Reads (×106) | Mapping Ratio (%) | All Gene Number |
| --- | --- | --- | --- | --- | --- | --- |
| Da 10-S1 | Da 10-S1-1 | 38.72 | 25.78(66.59%) | 26.57 | 68.62% | 19080 |
| | Da 10-S1-2 | 42.24 | 27.93(66.12%) | 28.77 | 68.12% | 19138 |
| | Da 10-S1-3 | 41.28 | 27.51(66.65%) | 28.35 | 68.68% | 19061 |
| Da 10-S2 | Da 10-S2-1 | 44.82 | 29.44(65.68%) | 30.41 | 67.87% | 18194 |
| | Da 10-S2-2 | 55.87 | 36.19(64.78%) | 37.43 | 67.01% | 18389 |
| | Da 10-S2-3 | 43.51 | 28.53(65.59%) | 29.49 | 67.80% | 18099 |
| Da 10-S3 | Da 10-S3-1 | 50.06 | 32.42(64.77%) | 33.74 | 67.41% | 17156 |
| | Da 10-S3-2 | 41.68 | 26.88(64.48%) | 27.99 | 67.17% | 16806 |
| | Da 10-S3-3 | 44.98 | 29.21(64.96%) | 30.42 | 67.64% | 16848 |
| Baisang-S1 | Baisang-1-1 | 41.54 | 26.85(64.65%) | 27.74 | 66.78% | 18950 |
| | Baisang-1-2 | 34.58 | 22.55(65.22%) | 23.29 | 67.36% | 18924 |
| | Baisang-1-3 | 39.77 | 25.84(64.98%) | 26.68 | 67.10% | 18940 |
| Baisang-S2 | Baisang-2-1 | 33.97 | 22.03(64.86%) | 22.72 | 66.90% | 18433 |
| | Baisang-2-2 | 36.28 | 23.35(64.37%) | 24.07 | 66.35% | 18388 |
| | Baisang-2-3 | 37.50 | 24.20(64.52%) | 24.94 | 66.51% | 18378 |
| Baisang-S3 | Baisang-3-1 | 41.97 | 27.45(65.41%) | 28.25 | 67.31% | 17457 |
| | Baisang-3-2 | 73.93 | 47.43(64.16%) | 48.81 | 66.03% | 18367 |
| | Baisang-3-3 | 33.52 | 21.80(65.02%) | 22.41 | 66.88% | 17438 |
