# Supplementary material for: Comparative transcriptome analysis of mulberry reveals anthocyanin biosynthesis mechanisms in black (Morus atropurpurea Roxb.) and white (Morus alba L.) fruit genotypes
Source: BMC Plant Biol. 2020 Jun 17;20:279. doi: 10.1186/s12870-020-02486-1 (PMC7301479; doi:10.1186/s12870-020-02486-1)
Supplement: Supplementary file 2 — Additional file 2: Table S2. Core genes related to anthocyanin biosynthesis in mulberry fruit. [file 12870_2020_2486_MOESM2_ESM.pptx]

## Slide 1
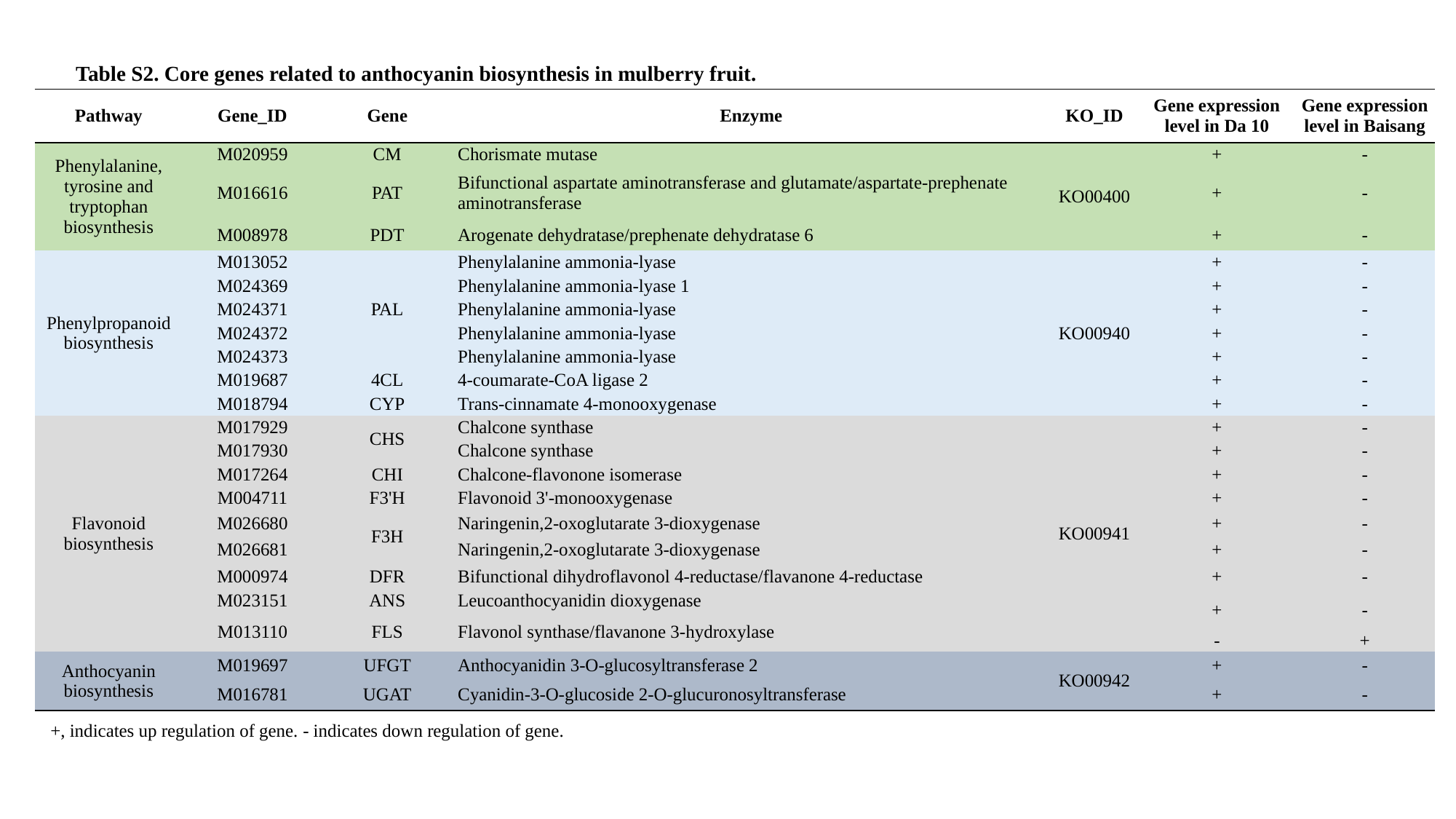

Table S2. Core genes related to anthocyanin biosynthesis in mulberry fruit.
| Pathway | Gene\_ID | Gene | Enzyme | KO\_ID | Gene expression level in Da 10 | Gene expression level in Baisang |
| --- | --- | --- | --- | --- | --- | --- |
| Phenylalanine, tyrosine and tryptophan biosynthesis | M020959 | CM | Chorismate mutase | KO00400 | + | - |
| | M016616 | PAT | Bifunctional aspartate aminotransferase and glutamate/aspartate-prephenate aminotransferase | | + | - |
| | M008978 | PDT | Arogenate dehydratase/prephenate dehydratase 6 | | + | - |
| Phenylpropanoid biosynthesis | M013052 | PAL | Phenylalanine ammonia-lyase | KO00940 | + | - |
| | M024369 | | Phenylalanine ammonia-lyase 1 | | + | - |
| | M024371 | | Phenylalanine ammonia-lyase | | + | - |
| | M024372 | | Phenylalanine ammonia-lyase | | + | - |
| | M024373 | | Phenylalanine ammonia-lyase | | + | - |
| | M019687 | 4CL | 4-coumarate-CoA ligase 2 | | + | - |
| | M018794 | CYP | Trans-cinnamate 4-monooxygenase | | + | - |
| Flavonoid biosynthesis | M017929 | CHS | Chalcone synthase | KO00941 | + | - |
| | M017930 | | Chalcone synthase | | + | - |
| | M017264 | CHI | Chalcone-flavonone isomerase | | + | - |
| | M004711 | F3'H | Flavonoid 3'-monooxygenase | | + | - |
| | M026680 | F3H | Naringenin,2-oxoglutarate 3-dioxygenase | | + | - |
| | M026681 | | Naringenin,2-oxoglutarate 3-dioxygenase | | + | - |
| | M000974 | DFR | Bifunctional dihydroflavonol 4-reductase/flavanone 4-reductase | | + | - |
| | M023151 | ANS | Leucoanthocyanidin dioxygenase | | + | - |
| | M013110 | FLS | Flavonol synthase/flavanone 3-hydroxylase | | | |
| | M013110 | FLS | Flavonol synthase/flavanone 3-hydroxylase | | - | + |
| Anthocyanin biosynthesis | M019697 | UFGT | Anthocyanidin 3-O-glucosyltransferase 2 | KO00942 | + | - |
| | M016781 | UGAT | Cyanidin-3-O-glucoside 2-O-glucuronosyltransferase | | + | - |
+, indicates up regulation of gene. - indicates down regulation of gene.
