# Supplementary material for: Comparative transcriptome analysis of mulberry reveals anthocyanin biosynthesis mechanisms in black (Morus atropurpurea Roxb.) and white (Morus alba L.) fruit genotypes
Source: BMC Plant Biol. 2020 Jun 17;20:279. doi: 10.1186/s12870-020-02486-1 (PMC7301479; doi:10.1186/s12870-020-02486-1)
Supplement: Supplementary file 3 — Additional file 3: Table S3. Primers used for qPCR. [file 12870_2020_2486_MOESM3_ESM.pptx]

## Slide 1
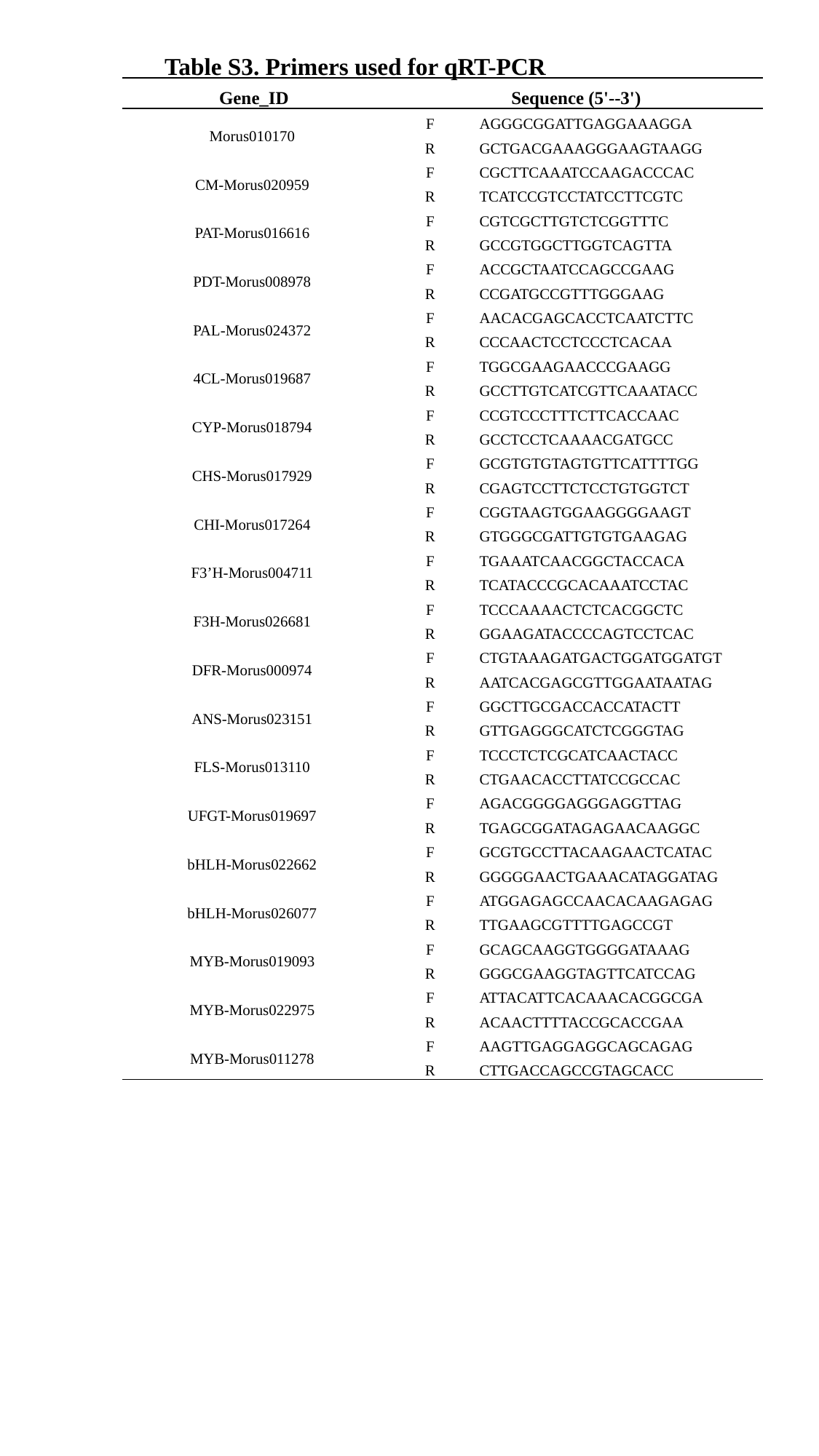

Table S3. Primers used for qRT-PCR
| Gene\_ID | | Sequence (5'--3') |
| --- | --- | --- |
| Morus010170 | F | AGGGCGGATTGAGGAAAGGA |
| | R | GCTGACGAAAGGGAAGTAAGG |
| CM-Morus020959 | F | CGCTTCAAATCCAAGACCCAC |
| | R | TCATCCGTCCTATCCTTCGTC |
| PAT-Morus016616 | F | CGTCGCTTGTCTCGGTTTC |
| | R | GCCGTGGCTTGGTCAGTTA |
| PDT-Morus008978 | F | ACCGCTAATCCAGCCGAAG |
| | R | CCGATGCCGTTTGGGAAG |
| PAL-Morus024372 | F | AACACGAGCACCTCAATCTTC |
| | R | CCCAACTCCTCCCTCACAA |
| 4CL-Morus019687 | F | TGGCGAAGAACCCGAAGG |
| | R | GCCTTGTCATCGTTCAAATACC |
| CYP-Morus018794 | F | CCGTCCCTTTCTTCACCAAC |
| | R | GCCTCCTCAAAACGATGCC |
| CHS-Morus017929 | F | GCGTGTGTAGTGTTCATTTTGG |
| | R | CGAGTCCTTCTCCTGTGGTCT |
| CHI-Morus017264 | F | CGGTAAGTGGAAGGGGAAGT |
| | R | GTGGGCGATTGTGTGAAGAG |
| F3’H-Morus004711 | F | TGAAATCAACGGCTACCACA |
| | R | TCATACCCGCACAAATCCTAC |
| F3H-Morus026681 | F | TCCCAAAACTCTCACGGCTC |
| | R | GGAAGATACCCCAGTCCTCAC |
| DFR-Morus000974 | F | CTGTAAAGATGACTGGATGGATGT |
| | R | AATCACGAGCGTTGGAATAATAG |
| ANS-Morus023151 | F | GGCTTGCGACCACCATACTT |
| | R | GTTGAGGGCATCTCGGGTAG |
| FLS-Morus013110 | F | TCCCTCTCGCATCAACTACC |
| | R | CTGAACACCTTATCCGCCAC |
| UFGT-Morus019697 | F | AGACGGGGAGGGAGGTTAG |
| | R | TGAGCGGATAGAGAACAAGGC |
| bHLH-Morus022662 | F | GCGTGCCTTACAAGAACTCATAC |
| | R | GGGGGAACTGAAACATAGGATAG |
| bHLH-Morus026077 | F | ATGGAGAGCCAACACAAGAGAG |
| | R | TTGAAGCGTTTTGAGCCGT |
| MYB-Morus019093 | F | GCAGCAAGGTGGGGATAAAG |
| | R | GGGCGAAGGTAGTTCATCCAG |
| MYB-Morus022975 | F | ATTACATTCACAAACACGGCGA |
| | R | ACAACTTTTACCGCACCGAA |
| MYB-Morus011278 | F | AAGTTGAGGAGGCAGCAGAG |
| | R | CTTGACCAGCCGTAGCACC |
