# Supplementary material for: Comparative transcriptome analysis of mulberry reveals anthocyanin biosynthesis mechanisms in black (Morus atropurpurea Roxb.) and white (Morus alba L.) fruit genotypes
Source: BMC Plant Biol. 2020 Jun 17;20:279. doi: 10.1186/s12870-020-02486-1 (PMC7301479; doi:10.1186/s12870-020-02486-1)
Supplement: Supplementary file 4 — Additional file 4: Figure S1. Biosynthesis pathway in mulberry fruit. a. Phenylalanine, tyrosine, and tryptophan biosynthesis. b. Phenylpropanoid biosynthesis. c. Flavonoid biosynthesis. d. Anthocyanin biosynthesis. [file 12870_2020_2486_MOESM4_ESM.pptx]

## Slide 1
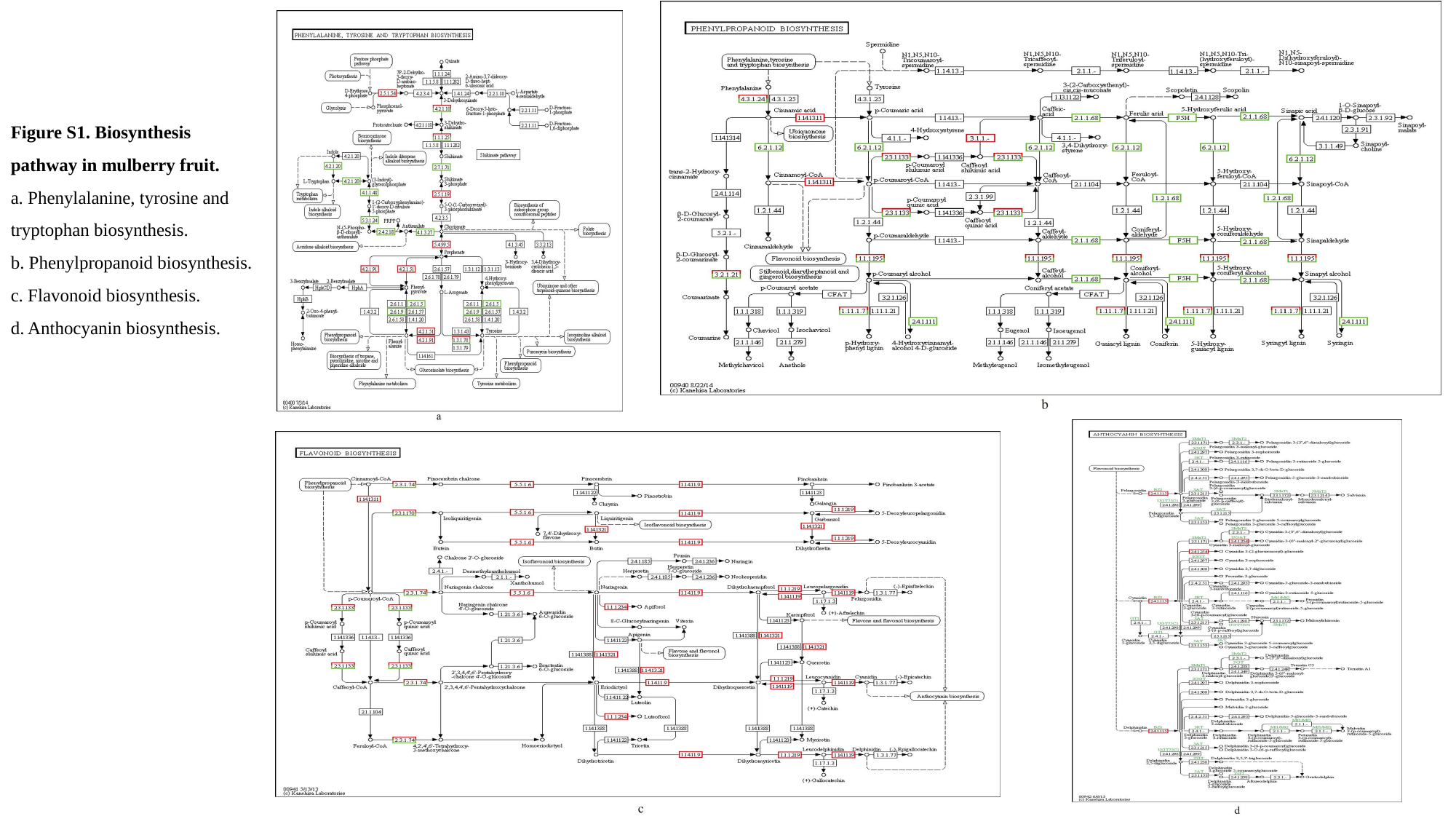

Figure S1. Biosynthesis pathway in mulberry fruit.
a. Phenylalanine, tyrosine and tryptophan biosynthesis.
b. Phenylpropanoid biosynthesis.
c. Flavonoid biosynthesis.
d. Anthocyanin biosynthesis.
