# Supplementary material for: Comparative transcriptome analysis of mulberry reveals anthocyanin biosynthesis mechanisms in black (Morus atropurpurea Roxb.) and white (Morus alba L.) fruit genotypes
Source: BMC Plant Biol. 2020 Jun 17;20:279. doi: 10.1186/s12870-020-02486-1 (PMC7301479; doi:10.1186/s12870-020-02486-1)
Supplement: Supplementary file 5 — Additional file 5: Figure S2. Expression levels of the core genes and transcription factors involved in anthocyanin biosynthesis in mulberry fruit, measured by qPCR. [file 12870_2020_2486_MOESM5_ESM.pptx]

## Slide 1
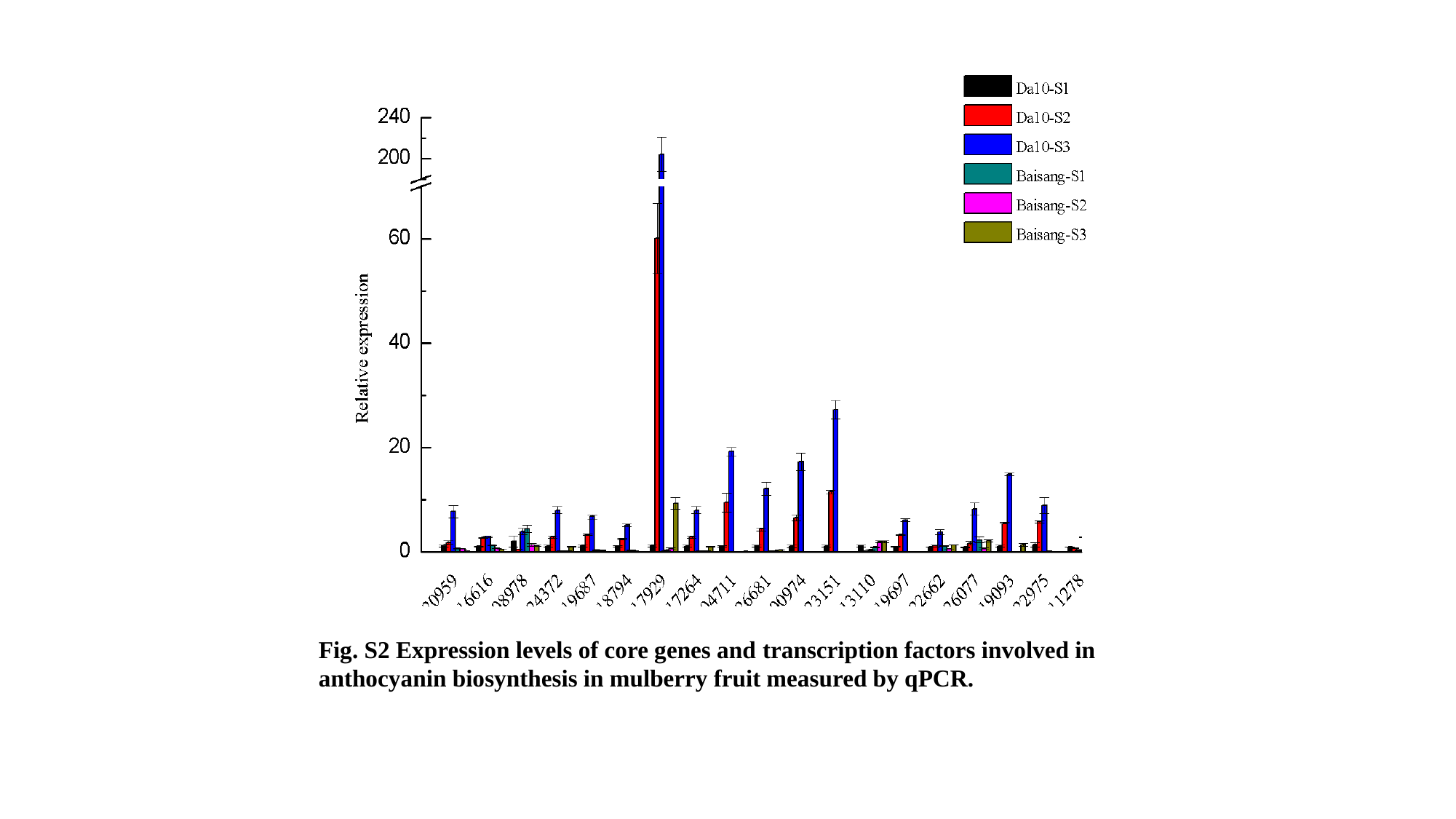

Fig. S2 Expression levels of core genes and transcription factors involved in anthocyanin biosynthesis in mulberry fruit measured by qPCR.
